# Randomized Trials Fit for the 21st Century. A Joint Opinion from the European Society of Cardiology, American Heart Association, American College of Cardiology, and the World Heart Federation

**DOI:** 10.5334/gh.1178

**Published:** 2022-12-16

**Authors:** Louise Bowman, Franz Weidinger, Michelle A. Albert, Edward T. A. Fry, Fausto J. Pinto, for the Clinical Trial Expert Group and ESC Patient Forum

**Affiliations:** 1 Clinical Trial Service Unit and Epidemiological Studies Unit, Nuffield Department of Population Health, University of Oxford, Oxford, UK; 2 Medical Research Council Population Health Research Unit, Nuffield Department of Population Health, University of Oxford, Oxford, UK; 3 European Society of Cardiology, 2nd Medical Department with Cardiology and Intensive Care Medicine, Klinik Landstrasse, Vienna, AT; 4 American Heart Association. Walter A. Haas-Lucie Stern Endowed Chair in Cardiology and Admissions Dean, University of California San Francisco Medical School. Director, CeNter for the StUdy of AdveRsiTy and CardiovascUlaR DiseasE (NURTURE Center), San Francisco, CA, US; 5 American College of Cardiology, Washington, DC, USA; 6 Ascension Health Cardiovascular Service Line, Indianapolis, IN, US; 7 World Heart Federation, Geneva, CH; 8 Department of Cardiology, Santa Maria University Hospital, CHULN E.P.E., CCUL, University of Lisbon, Lisbon, PT

## Graphical abstract

**Figure:**
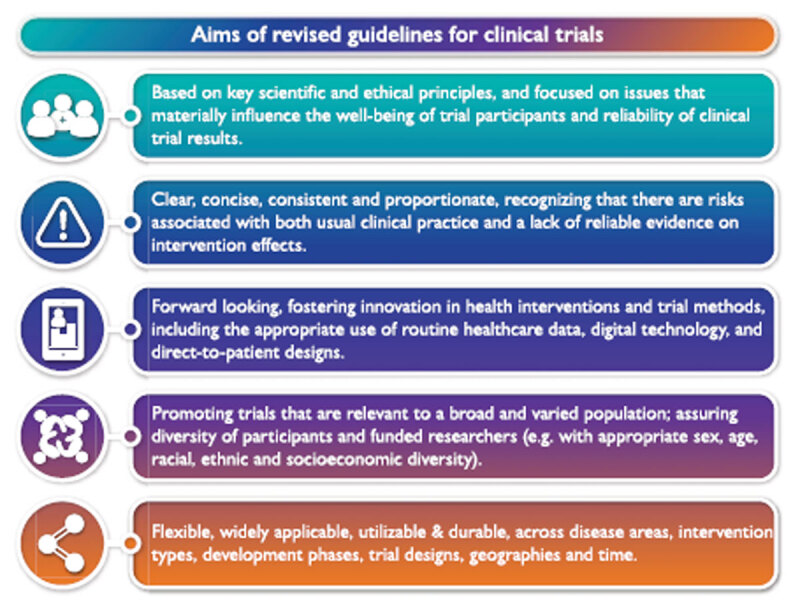


The views expressed in this article are those of the authors and therefore do not necessarily reflect the respective policies of the European Society of Cardiology, the American Heart Association, Inc., the American College of Cardiology, or the World Heart Federation.

## Problem

Randomized controlled trials are the cornerstone for reliably evaluating therapeutic strategies [1]. However, during the past 25 years, the rules and regulations governing randomized trials and their interpretation have become increasingly burdensome [2], and the cost and complexity of trials has become prohibitive [3]. The present model is unsustainable, and the development of potentially effective treatments is often stopped prematurely on financial grounds, while existing drug treatments or non-drug interventions (such as screening strategies or management tools) may not be assessed reliably. The current ‘best regulatory practice’ environment, and a lack of consensus on what that requires, too often makes it unduly difficult to undertake efficient randomized trials able to provide reliable evidence about the safety and efficacy of potentially valuable interventions. Inclusion of underrepresented population groups and lack of diversity also remain among the challenges.

The widespread availability of large-scale, population-wide, ‘real world data’ is increasingly being promoted as a way of bypassing the challenges of conducting randomized trials. Yet, despite the small random errors around the estimates of the effects of an intervention that can be yielded by analyses of such large datasets, non-randomized observational analyses of the effects of an intervention should not be relied on as a substitute, due to their potential for systematic error [4]. That is, the estimated effects may be precise but inaccurate, due to design and statistical biases that cannot be reliably avoided irrespective of the sophistication of the analysis.

With this joint opinion, the European Society of Cardiology (ESC), American Heart Association (AHA), World Heart Federation (WHF), and American College of Cardiology (ACC) call for action at a global scale to reinvent randomized clinical trials to be fit for purpose in the 21st century.

## Background

Among all medical specialities, cardiology has historically led the way in evidence-based practice. With ground-breaking randomized trials in the 1980s, such as the International Study of Infarct Survival (ISIS) [5], Gruppo Italiano per lo Studio della Streptochinasi nell’Infarto (GISSI) [6] and Global Utilization of Streptokinase and Tissue Plasminogen Activator for Occluded Coronary Arteries (GUSTO) [7] trials in acute myocardial infarction, cardiovascular ‘mega-trials’ were conceived and rapidly transformed clinical practice. High quality trials have also reliably demonstrated incremental clinical benefits with modification of major cardiovascular risk factors, such as hypertension [8] and dyslipidaemia [9], saving millions of lives worldwide in recent decades. Despite these advances, cardiovascular disease remains the leading cause of death and disability globally [10], and there is a need to identify additional effective therapies, to increase upstream prevention and precision medicine efforts, and to determine how best to use the effective treatments that we already have (and, as a corollary, not use those that are not effective or safe).

As age-specific rates of mortality and major morbidity decline due to better prevention and treatment, it becomes more difficult to conduct reliable assessments of new or existing interventions. Lower absolute risks of cardiovascular events mean that increasingly large samples are needed to generate the numbers of outcomes of interest, given the typically modest relative benefits of many interventions. Moreover, cardiovascular interventions often require sufficient time before the benefits emerge. As the size of trials increases, the cost rises, and there may be a temptation to limit the duration of follow-up, in order both to control costs and, from an industry perspective, to get new agents to market faster. The proprotein convertase subtilisin–kexin type 9 (PCSK9) inhibiting monoclonal antibodies (evolocumab and alirocumab) provide a recent example of such a strategy failing patients [11,12]. These agents have an impressive LDL cholesterol-lowering effect and, in large phase 3 randomized trials, were clearly shown to safely reduce major cardiovascular events. However, with only around 2–3 years of follow-up, it is likely that those trials underestimated the full benefits of prolonged PCSK9 inhibition on cardiovascular mortality and morbidity. So, despite the conduct of large trials which cost billions of dollars, the uptake of these agents has been limited (exacerbated by their high cost), and they have not realized their full potential for population health benefit even in high income countries.

During the past 25 years, there has been an enormous increase in the rules and related bureaucracy governing clinical trials. First issued in 1996, the International Council for Harmonization (ICH) Good Clinical Practice (GCP) Guidelines [13] describe the responsibilities and expectations of all those involved in the conduct of clinical trials. The intention of the ICH-GCP guideline was to ensure the safety and rights of participants in trials and also to ensure the reliability of trial results so that the safety of future patients would be protected. However, despite these well-intended aims, the guideline is now often over-interpreted and implemented in ways that are unnecessarily obstructive [14], prohibiting good trials from being done affordably. These problems are exacerbated by the financial incentive for some parties (in particular contract research organizations) to over-interpret ICH-GCP and profit from additional, often unnecessary, clinical trial procedures (such as frequent on-site monitoring visits when less costly data-driven monitoring approaches can be more informative [https://ctti-clinicaltrials.org/ourwork/quality/quality-by-design/]).

While the increasing complexities have been obstacles to trials conducted by industry, the regulations have become much larger barriers for conducting trials of interventions that have little or no commercial support. Consequently, trials of important questions relevant to low-income populations (e.g. infections affecting the heart such as rheumatic heart disease, tuberculous pericarditis or Chagas disease) or those that may have the potential for large clinical and population benefits but involve generic drugs (e.g. a polypill) have been hard to conduct.

## Opportunity for global impact

### Streamline the trial processes: reinvent simple trials with global impact

The COVID-19 pandemic has provided clinical trialists with an opportunity to rethink their trade and remember the landmark successes of the cardiovascular mega-trial concept established in the 1980s. Trials such as Randomised Evaluation of COVID-19 Therapy (RECOVERY) [15] and World Health Organization Solidarity [16] have been highly streamlined and designed to be easy to administer in the busy hospitals in which large numbers of COVID patients were being treated. Only essential data were to be collected and, wherever possible, much of the follow-up information was derived from national electronic health records (EHRs). Importantly, they showed that such trials can be conducted in accordance with the principles of GCP, but without over-interpretation or unnecessary complication. By contrast, many of the other COVID-19 trials had complex protocols (e.g. more restrictive eligibility criteria, significant additional data collection beyond that collected for routine care) with a focus on surrogate outcomes (e.g. time to clinical improvement, rather than mortality), such that their relatively small size did not allow them to yield clear evidence on the outcomes that matter most to patients [17,18]. Indeed, putative benefits observed in many small trials have not translated into mortality benefits when assessed in the larger streamlined trials [19].

### Use routine data to our advantage in trials, not as an inappropriate replacement

Considerable opportunities for streamlined trial conduct are provided by digital healthcare in the 2020s, with high quality EHRs available for both recruitment and follow-up of trial participants [20]. Part of the success of the RECOVERY trial was the nationwide availability of routine health data for comprehensive and complete follow-up. For many years, cardiovascular trials have successfully exploited EHRs for both recruitment and follow-up [as for example, in the Swedish Web-system for Enhancement and Development of Evidence-based care in Heart disease Evaluated According to Recommended Therapies (SWEDEHEART) series of trials], with important clinical findings [21]. Current initiatives are extending this approach through development and use of local and national registries that can facilitate low-cost, pragmatic ‘randomized registry trials’ [22]. However, data access restrictions and regulatory authority reticence to accepting EHR-based outcome data in randomized trials (especially for drug registration) have led to an underuse of this approach to trial streamlining. Instead, inappropriate emphasis is being placed—including by regulators—on using so-called ‘real world’ observational studies, despite the potential biases inherent in such methods.

### Collaborative revision of ICH-GCP, making it Fit for purpose in the 21st century

Recent experience has shown that important clinical questions can be addressed rapidly in streamlined trials while remaining compliant with existing guidelines. However, the approach taken to the implementation of the ICH-GCP guidelines is typically inflexible and frequently involves over-interpretation that stifles innovation in the clinical trials enterprise, driving up costs through waste, delay and failure. In consultation with a range of stakeholders—from patients and the public who volunteer for clinical trials, to organizations that provide the skills, funding and infrastructure to conduct research—the Good Clinical Trials Collaborative (GCTC https://www.goodtrials.org/) has been established by Wellcome, the Gates Foundation and the African Academy of Sciences to build on the work of the FDA-funded Clinical Trials Transformation Initiative (CTTI, https://ctti-clinicaltrials.org/) by producing comprehensive revised guidelines fit for the purposes of doing randomized trials in the 21st century. The GCTC is reviewing the principles for all types of healthcare interventions, in all settings, to produce guidelines that aim to foster and promote informative, ethical and efficient randomized controlled trials (see Graphical Abstract). Draft guidance was published for consultation and review in 2021, and it is anticipated that revised guidelines will be issued in 2022 (https://www.goodtrials.org/guidance).

We strongly support the adoption of this guidance into regulation, guidance, and practice across the whole clinical trials ecosystem—including by regulators, sponsors, and healthcare and research organizations—to ensure that the principles are embedded across all aspects of clinical trial design, delivery, oversight, quality assurance, analysis, and interpretation. Professional societies and their members have a key role to play in providing training in the fundamental principles of clinical trials, recognizing contribution to clinical trials as a core clinical activity, ensuring diversity and representativeness of included participants, and building community trust in the research enterprise by considering the patient perspective throughout all stages of trial development.

(https://nap.nationalacademies.org/catalog/26349/envisioning-a-transformed-clinical-trials-enterprise-for-2030-proceedings-of).

## Addressing the challenges: the role of cardiovascular organizations, societies, and foundations

Cardiology provided the foundation for an era of highly successful clinical trials, and is well-placed to reinvent trials for the 21st century. The ESC, AHA, ACC, and WHF are committed to ensuring that high quality trials continue to provide randomized evidence that improves the clinical care of all patients across different race and gender identities, socioeconomic strata, and geographies.

Technology has transformed medical practice in recent decades, and clinical trials need to keep pace if modern therapies and treatment strategies are to continue to be robustly evaluated. Digital advances provide streamlined solutions to trial conduct, such as app-based data collection, remote monitoring, and ‘virtual’ trial visits. The COVID-19 pandemic has forced us to think more critically about many elements of daily life with a rapid change in what is now considered ‘normal’. A timely opportunity exists to promote similarly radical changes into the conduct of trials, to enhance efficiencies while maintaining safety.

The cardiovascular organizations, societies, and foundations provide a valuable forum to advocate for the appropriate use of routine EHRs (i.e. ‘real world’ data) within randomized trials, recognizing the huge potential of centrally or regionally-held electronic health data for trial recruitment and follow-up, as well as to highlight the severe limitations of using observational analyses when the purpose is to draw causal inference about the risks and benefits of an intervention. With this document, our societies wish to engage in the development and widespread adoption of consensus guidance for clinical trials, supporting a more effective regulatory environment and allowing researchers to conduct the trials that are needed to improve patient care much more efficiently.

Finally, the COVID-19 pandemic has re-emphasized the importance of making it feasible for busy clinicians, and their patients, to participate in randomized trials. Without sustained efforts to increase the application of streamlined approaches, and a more supportive regulatory environment for those who do choose to generate randomized evidence (instead of the adversarial approach that is often taken in regulatory audits), patients will suffer from important clinical questions not being addressed reliably, either because trials are too small or, due to excessive financial or bureaucratic obstacles, are never done at all.

## Members of the Clinical Trial Expert Group

Stephan Achenbach, Department of Cardiology, Friedrich-Alexander, University Erlangen-Nürnberg, Erlangen, Germany; Louise Bowman, Nuffield Department of Population Health, University of Oxford, UK; Barbara Casadei, RDM, Division of Cardiovascular Medicine, NIHR Oxford Biomedical Research Centre, University of Oxford, UK; Rory Collins, Nuffield Department of Population Health, University of Oxford, UK; Philip J. Devereaux, Department of Medicine, McMaster University, Hamilton, Canada; Population Health Research Institute, Hamilton, Canada; Department of Health Research Methods, Evidence, and Impact, Canada; Pamela S. Douglas, Department of Medicine, Duke University School of Medicine, Durham, North Carolina, USA; Ole Frobert, Örebro University, Faculty of Health, Department of Cardiology, Örebro, Sweden; Department of Clinical Medicine, Aarhus University Health, Aarhus, Denmark; Shinya Goto, Department of Medicine (Cardiology), Tokai University School of Medicine, Isehara, Japan; Cindy Grines, Northside Hospital Cardiovascular Institute, Atlanta, Georgia, USA; Robert A. Harrington, Department of Medicine, Division of Cardiovascular Medicine, Stanford University, CA, USA; Richard Haynes, MRC Population Health Research Unit, Nuffield Department of Population Health, University of Oxford, UK; Judith S. Hochman, Leon H. Charney Division of Cardiology, Department of Medicine, New York University Grossman School of Medicine, New York, USA; Stefan James, Uppsala Clinnical Research Center and Department of Medical Sciences, Uppsala University, Uppsala, Sweden; Paulus Kirchhof, Department of Cardiology, University Heart and Vascular Center Hamburg, University Medical Center Hamburg Eppendorf, Germany; Atrial Fibrillation Competence NETwork (AFNET), Münster, Germany; Institute of Cardiovascular Sciences, University of Birmingham, UK; Michel Komajda, Department of Cardiology, Groupe Hospitalier Paris Saint Joseph, Sorbonne University, Paris, France; Carolyn S.P. Lam, National Heart Centre Singapore & Duke-National University of Singapore, Singapore; Martin Landray, Nuffield Department of Population Health, University of Oxford, UK; Aldo Maggioni, ANMCO Research Centre, Florence, Italy; John McMurray, British Heart Foundation Cardiovascular Research Centre, Institute of Cardiovascular & Medical Sciences; University of Glasgow, UK; Nick Medhurst, Good Clinical Trials Collaborative https://www.goodtrials.org/; Roxana Mehran, Icahn School of Medicine at Mount Sinai, New York, USA; Bruce Neal, The George Institute for Global Health, University of New South Wales, Sydney, Australia; School of Public Health, Imperial College London, London, UK; Lars Rydén, Department Medicine K2, Karolinska Institutet, Stockholm, Sweden; Holger Thiele, Heart Center Leipzig at University of Leipzig and Leipzig Heart Institute, Department of Internal Medicine/Cardiology, Leipzig, Germany; Isabelle Van Gelder, University Medical Center Groningen, University of Groningen, Groningen, The Netherlands; Lars Wallentin, Uppsala Clinnical Research Center and Department of Medical Sciences, Uppsala University, Uppsala, Sweden; Salim Yusuf, Population Health Research Institute, McMaster University and Hamilton Health Sciences, Hamilton, ON, Canada; Faiez Zannad, Université de Lorraine, Inserm and CHRU, Nancy, France. The **ESC Patient Forum** https://www.escardio.org/The-ESC/What-we-do/esc-patient-engagement.
